# Adolescent Propensity to Engage in Health Risky Behaviors: The Role of Individual Resilience

**DOI:** 10.3390/ijerph7052161

**Published:** 2010-05-04

**Authors:** Mir M. Ali, Debra S. Dwyer, Elizabeth A. Vanner, Alexander Lopez

**Affiliations:** 1 Department of Economics, University of Toledo, Toledo, OH 43606, USA; 2 School of Health Technology & Management, Stony Brook University, Stony Brook, NY 11794-8204, USA; E-Mails: debra.dwyer@stonybrook.edu (D.S.D.); Elizabeth.vanner@stonybrook.edu (E.A.V.); alexander.lopez@stonybrook.edu (A.L.); 3 Department of Economics, Stony Brook University, Stony Brook, NY 11794-8204, USA; 4 School of Medicine, Stony Brook University, Stony Brook, NY 11794-8204, USA

**Keywords:** resilience, health risky behavior, factor analysis, adolescent health

## Abstract

In this paper we create indices of resilience to identify adolescents at risk of smoking, drinking alcohol, and using illegal drugs. Using data from the National Longitudinal Study of Adolescent Health, three manifestations of resilience were identified: overall-resilience, self/family-resilience, and self-resilience. Our analysis reveals that the overall-resilient were less likely to engage in risky behaviors. The self/family resilient were more likely to engage in risky behaviors, but consumed less. The self-resilient had reduced risk for smoking and drinking alcohol but elevated risk for using illegal drugs and being in an addictive stage of smoking and drinking, if participating.

## 1. Introduction

In its report, *Youth Risk Behavior Surveillance—United States 2005*, the CDC asserts that “health-risk behaviors, which contribute to the leading causes of morbidity and mortality among youth and adults, often are established during childhood and adolescence, extend into adulthood, are interrelated, and are preventable [1]. Results from the CDC’s national Youth Risk Behavior Survey 2005 indicated that, during the prior 30 days, 23.0% had smoked cigarettes, 43.3% had consumed alcohol, 20.2% had used marijuana, and 2.1% reported past injection of an illegal drug [1]. Risky behavior among adolescents has decreased since 1991, but many still engage in behaviors which contravene achieving the goals of Healthy People 2010 [2]. Those who continue to engage despite policy interventions may be most at risk for negative outcomes.

The CDC calls for “more effective school health programs and other policy and programmatic interventions which are needed to reduce risk and improve health outcomes among youth” [1]. While school health programs may have some impact, it is critical for such programs to target those adolescents most at-risk. This research seeks to increase the effectiveness and target efficiency of health policies and programs whose aim is to reduce health-risk behaviors among adolescents, by exploring the role of resilience in adolescents’ risk for participation in these behaviors.

Efforts have been made to identify processes of both vulnerability and protection to reduce health compromising behaviors in adolescents [3]. Resilience refers to an individual’s capacity to engage in health-promoting behaviors in the face of adversity [4–9] and is an important element in human adaptation. It is a distinguishing quality necessary for adapting to changing demands of daily living. Work in psychology [5,7,8,10–12] introduces the notion of resilience to understand coping and adaptation, which can influence activity preferences, and supports the premise that resilient adolescents are more able to endure stress and avoid addiction [5,7,8]. Two individuals facing the same family and social pressures may respond differently based on unobserved attributes like resilience. Previous literature [5,7,13] has suggested that factors associated with resilience and vulnerability occur on three levels: personal, family, and community and that each contributes to an individual’s overall resilience. Social supports outside the family contribute to community-level resilience, family-level resilience is associated with parental function and relationships, and personal-resilience is associated with the individual’s psychological, cognitive, and emotional processes [5,7,8,13,14]. The indices we construct in this paper capture resilience in these areas. While an individual’s stock of resilience at any given point in time is also a function of these outside influences (community and family), it is the perception of one’s own situation that will ultimately drive behaviors such as smoking, drinking and drug use. There are three models of resilience in the literature. In this paper we build on the compensatory model of resilience that posits that the collective appraisals of risk and compensatory factors predict competence. For example, negative personal self image (risk factor) combined with a positive perception of peer support, respect and admiration (compensatory factor) may have a neutralizing effect on an individual’s propensity to engage in unhealthy activities [15–18]. Whether the factor is risky or compensatory is determined by the individual’s own self perception. We construct our index in line with this theoretical framework. The other two models provide alternative rationale for resilience in adolescents. The protective factor model identifies social and environmental assets and resources as factors that reduce the degree of risk [19]. The challenge model [18] focus on risk factors as facilitators of adaptation. Individuals build on their resilience capacities when confronted with stress that does not exceed their ability to cope.

Indications of not enjoying life, feeling sad, feeling hopelessness *etc*., imply lower personal competence and resilience. Alternatively, feeling one has good qualities and having a sense of accomplishment and pride are indicators of a healthy sense of personal competence and mastery and, consequently, of resilience [4,6,20–23]. In this research, we use indicators of these emotional attributes to construct measures of resilience in adolescents.

An adolescent functions in some dominant culture with customs, practices, ideologies, values, norms, and beliefs. The social environment, in addition to personal competence, is fundamental to fostering and exercising resilience, which, in adolescents, may be influenced by adverse experiences [13,24,25]. The social context might afford opportunities for feeling socially accepted, loved, and wanted (helping develop an adolescent’s resilience), or, conversely, might foster a sense of vulnerability, hindering development of resilience and resulting in initiation of activities that compromise health [6].

Adolescents engage in activities that satisfy their physiological and psychological needs and provide physical and mental comfort and security. The activities chosen (health-promoting or health-compromising) depend on the contexts in which the individual exists. Regardless of the activities’ benefits or detriments, engagement in these activities is contextually purposeful and meaningful to the individual [26]. Therefore, an adolescent’s overall resilience is not just a function of individual makeup, but also strongly impacted by social constructs. We also use indicators of an adolescent’s contextual/social attributes in our construction of resilience measures.

Factors such as peer and family influences, school, and policy environment have all proven to be predictors of early initiation of risky products like cigarettes [27–29], and policies have been enacted to reduce initiation, but with mixed results. At the margin, most of these policies have had some impact. The economics literature consistently finds that increased price reduces initiation [30]. However, it may be that “sin taxes” and advocacy programs have disproportionately provided incentives and information to deter such behavior among adolescents for whom addiction was an unlikely outcome [31,32]. To effectively reduce initiation and addiction, current policies may not be optimal since their focus is on adolescents in general, rather than those with the highest propensity for adverse outcomes. For example, the D.A.R.E. (Drug Abuse Resistance Education) program, once offered by 75% of American school districts to all adolescents and/or pre-adolescents in certain grades, may be eliminated by some districts because studies have not demonstrated the program’s effectiveness [33–35]. Some districts are considering replacing D.A.R.E. with alternative programs which assess “the levels of risk factors related to problem behaviors such as alcohol, tobacco and other drug use—and to identify the levels of protective factors that help guard against those behaviors” [36].

We suggest that one difficulty in achieving success with existing anti-addiction policy is the lack of research that identifies at-risk groups. Jessor (1991) believes the aim of research regarding risky adolescent behavior should be identifying its psychosocial antecedents and determinants, that is, “What are the risk factors for the risk factors?” [37], and that effective policy in reducing this behavior must address an adolescent’s capacity for coping with life as a factor influencing initiation of risky behaviors [4,9,20]. Our premise is that the protective factors which contribute to resilience help adolescents endure stress and avoid addiction-associated psychosocial impairments [5,7,8]. By developing resilience indices, we identify an important source of heterogeneity among adolescents in their propensities to cope, in order to identify those that may be more or less at risk for participating in health-compromising behavior.

Policy makers need to know who is at risk, the magnitude of that risk, and the factors that drive risky behavior, to develop target efficient policies aimed at improving outcomes among adolescents. Considering personal, psychosocial, and environmental factors when developing policy to reduce risky behavior should enable interventions to reduce participation and improve health more efficiently. Further, if resilience is a key factor in predicting outcomes, effective policies may attempt to improve resilience among adolescents, strengthening coping skills and the contextual factors that contribute to resilience [38]. The three models of resilience in the literature can be used to inform policy. Therefore, the ability to assess resilience in adolescents might help predict who is at risk for addiction, enabling addiction prevention policies to be targeted at those adolescents most likely to benefit. Using factor analysis on data from the nationally representative sample of middle/high school students in the National Longitudinal Study of Adolescent Health [39] (Add Health), we identified three, mutually-exclusive manifestations of resilience. Consistent with assumptions and prior literature, these are related to social, family, and individual resilience [6]. To identify the effect of adolescent resilience on the risk of engaging in health-compromising behaviors (smoking, drinking alcohol, and using illegal drugs) and subsequent addiction, we tested the following hypothesis: Adolescents who are less resilient will be more likely to smoke, drink alcohol, and use illegal drugs and will consume greater quantities of cigarettes, alcohol, and illegal drugs.

## 2. Methods

### 2.1. Subjects

This study used data from the National Longitudinal Study of Adolescent Health [39] (Add Health), which consists of data on adolescents from 132 schools nationwide, between grades 7 through 12 in 1994. A description of the sample may be found in Table 1. The in-school portion of the first wave of the survey (1994) contains a cross-section of data on about 90,000 adolescents. A subset of the initial sample (20,745 respondents) was also interviewed in their homes with follow-up surveys in 1996 and in 2002. The primary data for our analysis comes from the first wave (1994) of the in-home survey portion of Add Health. The study benefits from the richness of the Add Health data, both in terms of sample size as well as the quality of the measures of typically unobserved school, peer, family, and individual attributes [13]. We capitalize on this strength to identify adolescents at risk. The use of the restricted secondary Add Health data has been approved by the Carolina Population Center.

The sample for our analysis included the 20,700 in-home sample adolescents who responded to all relevant questions for this study. Parents of the in-home sample were also interviewed regarding the parent-child relationship as well as parents’ smoking and alcohol consumption. Additionally, school administrators provided attributes of the school, including anti-smoking, -alcohol, and -drug policies/prevention programs.

### 2.2. Construction of Resilience Indices

There were 31 Add Health questions identified as being related to resilience (Table 2). Putting all 31 questions into the model would be noisy and difficult to interpret, particularly because of how collinear they are. Using exploratory factor analysis, a simple, yet efficient data reduction technique, these 31 questions were reduced to three, mutually-exclusive, underlying, latent indices (factors) [6] which significantly captured the heterogeneity in the way that resilience is manifested in adolescents. The methodology combines like underlying components of the 31 questions, and groups them into the three mutually exclusive indices. This minimizes the potential for attenuation bias that might result from indicators that may have offsetting effects (the attribute is associated with a good outcome, but may also have the potential to be associated with a negative outcome). Table 2 contains the factor loadings and scoring coefficients from the factor analysis that depicts how each indicator can have more than one non-random significant component that is tied to different underlying attributes of adolescent resilience.

Factors are typically selected based on the magnitude of the eigenvalues. Some use a cutoff of eigenvalue = 1 to retain a factor, while others argue that this is arbitrary, and that factors should be retained until there is a dramatic drop in the eigenvalue [40]. This latter approach is more consistent with the intent of factor analysis: an exploratory quest for key, underlying commonalities in multiple indicators. Based on analyzing the factor loadings, we retained a factor as long as it loaded onto at least one of the 31 questions stronger than the factors that preceded it. Had we used a cutoff of 1, we would have retained only the first two factors, throwing information away. The third factor (eigenvalue close to 0.9) had information that appeared to be significant and which was not contained in the other factors. We felt it preferable to retain a less important factor than risk throwing away a significant one, especially in light of the fact that the literature suggests that resilience has three separate aspects related to personal, family, and community [5–7].

Scoring high on Factor 1 (eigenvalue = 3.85, *M* = 0.248, *SD* = 0.887) indicated overall-resilience, with self, family, and peer/community components. Individuals scoring high on this factor averaged positive responses to most of the 31 questions. Scoring high on Factor 2 (eigenvalue = 1.46, *M* = 0.009, *SD* = 0.782) indicated self/family-resilience without peer/community components. We interpret strong sense of self from favorable responses to components such as being happy, hopeful, and optimistic and having good qualities. For this index we also interpret strong sense of family support because they felt loved, wanted and that their parents care. However, while this group reported feeling socially accepted and happy, they also were more likely to feel lonely, sad, and that people were unfriendly. This suggests some strong family ties, and perhaps some good friends, but that they struggle socially; perhaps these are the type who are part of a smaller group of friends that are not popular. Scoring high on Factor 3 (eigenvalue = 0.86, *M* = −0.007, *SD* = 0.705) indicated self-resilience without family and peer/community components. We retained this factor because it had the highest load onto being non-argumentative so it contained information not captured elsewhere. Respondents scoring high in this factor are a type that are worthy of study. While they report lack of support from parents and teachers, some of the areas they score high in suggest strength within themselves. For example these types score high on factors like not being sad, liking oneself, and confidence going with gut feelings, suggesting strong self in the absence of feeling that adults care about them. They were also less likely to face their problems—which could be a protective coping mechanism that keeps the adolescent optimistic, despite difficult circumstances. This type of adolescent has chosen to trust and rely on self given lack of alternatives.

If resilience in adolescents occurred such that the manifestation of any type of resilience (self, family, and/or community) was independent of the manifestation of any other type of resilience, then we would expect the factor analysis to reveal three distinct components of resilience: self alone, family alone, and community alone. However, the factor analysis revealed that, in adolescents, the manifestation of types of resilience is NOT independent, but rather highly correlated. One of the assumptions of factor analysis is that there is enough independence in the manifestation of distinct components so that the factor analysis can tease the components apart. Our results suggest that this assumption that self, family and community resilience are independent is violated. That is why the factor analysis results indicated three factors that are empirically (that is, based on the data) mutually-exclusive but not conceptually mutually exclusive, because, for example, clearly overall-resilience contains elements of family-resilience and self-resilience. The factor analysis did not find three mutually-exclusive types of resilience, but rather revealed, among adolescents, three mutually-exclusive manifestations of resilience: overall-resilient, self/family-resilient, or self-resilient. In other words, the factor analysis reveals three distinct types of adolescents that have some overlapping self-reported attributes. We can now identify these distinct types of adolescents in our model of behavior to better identify which types are at risk. A table showing the means and standard deviations for each sample characteristic (see Table 1 for a list of characteristics) for each of the three resilience factors (overall, self/family, and self) is available from the authors on request.

### 2.3. Constructing Participation and Consumption Indicators

Variables: Dependent variables included participation and consumption (smoking, drinking, or using illegal drugs). Independent variables included resilience, other personal factors, family relations, peer behavior [41,42], and school policies. Confounding variables included parental education, and whether or not the adolescent lived with both biological parents, in an urban setting, at least one parent smoked, the parents consumed alcohol, and the adolescent indicated easy access to cigarettes at home.

Smoking was classified into four levels: non- (never, ever tried cigarettes), experimental- (tried cigarettes, denied smoking within the past 30 days, never smoked regularly), intermittent- (smoked between 1 and 29 of the past 30 days), or regular-smoker (smoked on a daily basis the past 30 days) [43,44], with the latter three groups classified as participating. Consumption was the number of cigarettes smoked each day that the person smoked. Drinking was classified into three levels: non- (never drank alcohol), light/moderate- (having one to four drinks each time), or heavy-drinker (having five or more drinks each time), with the latter two groups classified as participating. Drinking consumption was the number of drinks the adolescent had each time they drank alcohol. Illegal drug use was classified into two levels with those who reported to have tried any illegal drugs in the previous 30 days categorized as participating and consumption was measured by number of times drugs were used in the last 30 days.

A variable was constructed, using exploratory factor analysis, to capture both the child’s and parent’s perception of the parent-child relationship, to reflect roles that parents play in influencing adolescents’ risky behavior [45]. Adolescents contributed perceptions of whether he/she thought that his/her parents care, understand, pay attention, *etc*. and parents contributed perceptions of whether he/she got along well with the child, could trust the child, *etc*. The factor analysis results indicated that a single factor (eigenvalue = 2.25837) was sufficient to summarize all variables in the parent-child relationship.

The average number of students in the respondent’s grade and school who smoked, consumed alcohol, or used illegal drugs was used to identify peer influence. To account for various school policies, models used school program indictors (whether the school offered alcohol, drug, and/or smoking prevention/awareness programs) and school restriction indictors (whether smoking was prohibited for students and faculty on the school premises and/or whether possession of drugs and/or alcohol resulted in suspension from school).

Data Analysis: Since the participation variables were binary (adolescents either participated or they did not participate), probit analysis was used to build three participation models for smoking, drinking, and using illegal drugs. Ordinary least-squares multiple regression was used to build three consumption models: quantity of cigarettes, alcohol, and illegal drugs. Besides controlling for observed environmental factors, we also controlled for unobserved differences within neighborhoods by introducing school-level variables (fixed effects) in all of our estimations. This allowed us to account for environmental differences at the school level that might have influenced resiliency at the individual level.

## 3. Results

The results of our analyses of the influence of resilience on risky behavior are reported in Table 3. The effect of resilience persisted even after controlling for family, peer, and other environmental and personal factors. For each behavior (smoking, drinking alcohol, and using illegal drugs) we report the difference in the probability of participating (columns 1, 9 and 17) from the average probability (for a marginal increase in the resilience index or other variable) and then, for those who do participate, the incremental amount consumed (columns 5, 13 and 21). We modeled consumption thus to understand severity of the behavior among those who chose to participate. (Overall consumption results, which are similar to these, are also available, upon request.)

A one unit increase in the overall-resilience index reduces the probability of smoking by almost 5%, by 6% for drinking, and by 4% for illegal drug use. Those who were self/family-resilient but perhaps are weaker in social resilience were at a higher risk for participation. Individuals who score marginally higher in this index are 6–7% more likely to smoke or drink and 2% more likely to use illegal drugs than the average. Those who were self-resilient were marginally *less* likely to smoke or drink, but 1% more likely to use illegal drugs.

For consumption (conditional on participation), those who were overall-resilient consumed less alcohol and cigarettes with no difference in illegal drug consumption. Those who were self-resilient consumed more cigarettes, while those who were self/family-resilient smoked fewer cigarettes. Drinkers who were self-resilient consumed above average quantities of alcohol. Among drug users (smallest sample), there was no difference in consumption by resilience.

The confounding factors were also significant in predictable ways for both participation and consumption. The role of parents was especially important with parental education and participation in risky behaviors, as well as the relationship with the child, all being significant factors. After controlling for resilience, older adolescents, males, whites, and Hispanics were more likely to engage in risky behavior. Religious adolescents were less likely to do so.

## 4. Discussion

The literature suggests three components of resilience (personal, family, and community) [8–11]. The data confirm three significant underlying components, with the bulk of what determines resilience captured by the first factor. However, two other factors, with sufficiently high eigenvalues and factor loadings, indicated residual commonalities worth including beyond the first resilience index. It was predicted that adolescents with high scores on these three factors had greater resilience and, in turn, would exhibit better outcomes than those with low scores, after controlling for other risk factors.

The results (see Table 3) indicate that self/family-resilience, in the absence of peer/community resilience, was inadequate to prevent participation in risky behaviors (Risk factors - smoking, drinking, and using drugs). Perhaps those who experience an absence of peer acceptance (compensatory factors) may be unable to resist risky behaviors due to peer pressure to “fit in.” However, strong self/family-resilience can lead to a reduction in the number of cigarettes smoked. These may be the experimenters or intermittent participants who smoke when out socially but with strong enough family support and self resilience to know when to stop. Alternatively, those who were overall-resilient may be better equipped to resist peer pressure to smoke, drink, or use drugs. Interestingly, those who were self-resilient, with little family or social support, were less likely to smoke or drink but those who did consumed larger quantities. In a model where we control for other risk factors like family characteristics, strong self resilience (coping skills) predictably reduces propensities to engage in such behaviors. There does appear to be a split among adolescents who fall into this resilience type since those who do participate are the target group at highest risk for addiction since among those who do participate, they are consuming closer to addictive levels. In addition, within this type are those who are more likely to turn to illegal drug use rather than face problems.

In sum, perhaps smokers who were overall-resilient were primarily experimental, smokers who were self/family-resilient were primarily experimental or intermittent (mainly to fit in socially), while smokers who were self-resilient were primarily regular smokers who may smoke as an escape, rather than for social acceptance. While those who were self-resilient consumed an average amount of drugs, they were more likely to use drugs, and any use of illegal drugs could be seeking an escape. These associations between resilience and consumption are particularly noteworthy because the consumption models contained only participants, who were more homogeneous in resilience and other risk factors, since these factors already significantly explained the propensity to participate.

Programs like the DARE program may have had little impact because they are not target efficient [27–29]. This work has implications for policies aimed at improving outcomes for adolescents at risk of engaging in the health compromising activities. School wide programs that are “one size fits all” may not yield desirable results without attempting to tailor the program to include incentives that will work for adolescents at risk. An alternative approach might be to identify at risk children in schools based on resilience scores and develop programs to increase self efficacy and compensate for lack of environmental assets and resources. Occupational therapists provide interventions in mental health and school-based settings that assist clients in developing compensatory strategies for building resilience and facilitating healthy adaptation. In school-based practice, occupational therapy can provide target specific interventions that meet the specific needs of at risk children and promote resilience [46].

One limitation of this study is that the Add Health data was not designed with the intent to construct indicators of resilience. So while the data are rich with indicators that overlap those used in the literature to construct resilience, the questions were not asked in a sequence consistent with how it might be done in a resilience study, and we had to pool together indicators throughout the survey piecemeal.

These results motivate further analysis of the relationships between adolescent resilience and risky behavior. We can further examine consumption findings, to test for level of addiction, such as whether those who were self-resilient were more likely to be regular smokers while those who were self/family-resilient were more likely to smoke intermittently. We also plan to further explore the psychometric properties of these resilience indices, comparing them to another measure of resilience and measures of related constructs (in another sample of adolescents) to assess concurrent and discriminant validity. Future research will also examine how resilience evolves over time and how this change affects addictive behavior. Data from future waves of Add Health (1996 and 2002) will be analyzed to further assess how adolescent resilience affects behavior throughout adult life. Also, interventions should be tested which are targeted toward vulnerable adolescents, engaging them in experiential learning and problem-solving to increase resilience and health-promoting behaviors when available personal, family, and peer/community resources are insufficient [47,48].

In conclusion, we hypothesized that adolescents vary in resilience and using a nationally representative sample of middle/high school students, we examined the role of individual resilience in predicting addictive behaviors: smoking, alcohol consumption, and illegal drug use. Examining addictive behavior in adolescents, controlling for confounding environmental and personal factors, the role of resilience was robust to various model specifications and significantly explains heterogeneous risks. We suggest that an adolescent’s propensity for detrimental risky behavior, and subsequent negative outcomes, relates to environmental and personal factors, including resilience. The value of knowing that resilience among adolescents tends to occur in one of three types: overall-resilient, self/family-resilient, or self-resilient, is that programs to reduce health-compromising behaviors can be more effective and efficient if they are targeted to adolescents with specific needs, which can be identified by their type of resilience.

## Figures and Tables

**Table 1.** Sample Characteristics.

Variable	number	percent
**Dependent**		
Cigarette Smoker		
Yes	11,139	53.69
No	9,606	46.31
Drinker of Alcohol		
Yes	9,646	46.50
No	11,099	53.50
Illegal Drug User		
Yes	2,933	14.14
No	17,812	85.86
**Demographic**		
Grade (1994)		
7	2,716	13.09
8	2,718	13.10
9	4,179	20.14
10	3,967	19.12
11	3,809	18.36
12	3,356	16.18
Religious (1994)		
Yes	8,913	42.96
No	11,832	57.04
Gender		
Male	10,482	50.53
Female	10,263	49.47
Race		
White	10,761	51.87
Black	4,633	22.33
Hispanic	3,431	16.54
Asian	1,584	7.64
Other	336	1.62
Urban (1994)		
Yes	9,025	43.50
No	11,720	56.50
**Parent**		
Parent Smoke		
Yes	15,738	75.86
No	5,007	24.14
Parent Drink		
Yes	7,959	38.37
No	12,786	61.63
Easy Access (cig.)		
Yes	14,246	68.67
No	6,499	31.33
Live w/both parents		
Yes	10,406	50.16
No	10,339	49.84
Mother’s Education		
< high school	4,094	19.73
HS, no college	11,552	55.69
College & above	5,099	24.58
Father’s Education		
< high school	3,085	14.87
HS, no college	13,302	64.12
College & above	4,358	21.01

**Table 2.** Add Health Questions with Factor Loadings and Scoring Coefficients to Construct Resilience Indices.

Question		Index 1 E=3.85		Index 2: E=1.46		Index 3: E=0.86	
	Resilience Type:	Overall		Self / Family		Self	
		Factor Loading	Scoring Coefficient	Factor Loading	Scoring Coefficient	Factor Loading	Scoring Coefficient
01. You felt that you could not shake off the blues, even with help from your family and your friends. (Not Blue : 0 – no ; 1 –yes).		**0.3304**	0.0624	**−0.1812**	−0.0710	−0.0467	−0.0270
02. You enjoyed life. (Life : 0 – no ; 1 – yes).		**0.2920**	0.0608	**0.2722**	**0.1181**	**−0.1302**	−0.0806
03. You felt sad. (Not Sad : 0 – no ; 1 –yes).		**0.4902**	**0.1193**	**−0.3673**	**−0.1873**	0.0463	0.0328
04. You felt that people disliked you. (Not Dislike : 0 – no ; 1 – yes).		**0.4719**	**0.1118**	**−0.2593**	**−0.1258**	−0.0886	−0.0643
05. You never argue with anyone. (Never Argue : 0 – no ; 1 – yes).		**0.1622**	0.0335	**−0.1212**	−0.0519	**0.3829**	**0.2239**
06. You never get sad. (Never Sad : 0 – no ; 1 – yes).		**0.2527**	0.0511	**−0.1919**	−0.0821	**0.3962**	**0.2478**
07. Difficult problem makes you very upset. (Problem not upset : 0 – no ; 1 – yes).		0.0848	0.0151	**−0.2009**	−0.0753	**−0.1632**	−0.0915
08. You were bothered by things that usually don't bother you. (Not Bother : 0 – no ; 1 – yes).		**0.4156**	0.0865	**−0.2769**	**−0.1191**	−0.0422	−0.0281
09. You felt that you were just as good as other people. (Good : 0 – no ; 1 – yes).		**0.2046**	0.0381	**0.1712**	0.0684	**−0.1626**	−0.0906
10. You had trouble keeping your mind on what you were doing. (Not troubled : 0 - no; 1 – yes).		**0.3707**	0.0781	**−0.2979**	**−0.1292**	−0.0446	−0.0286
11. You felt hopeful about the future. (Hopeful : 0 – no ; 1 – yes).		**0.1806**	0.0356	**0.2174**	0.0867	**−0.1659**	−0.0932
12. You thought your life had been a failure. (Not Failure : 0 – no ; 1 – yes).		**0.5226**	**0.1140**	−0.0712	−0.0299	**−0.1052**	−0.0732
13. You were happy. (Happy : 0 – no ; 1 – yes).		**0.2937**	0.0607	**0.2418**	**0.1043**	**−0.1216**	−0.0736
14. It was hard to get started doing things. (Not Hard : 0 – no ; 1 – yes).		**0.3253**	0.0637	**−0.2453**	**−0.1006**	−0.0093	−0.0058
15. When you get what you want, it’s usually because you worked hard for it. (Work Hard : 0 – no ; 1 – yes).		**0.2062**	0.0366	**0.1265**	0.0455	0.0601	0.0292
16. You usually go out of your way to avoid having to deal with problems in your life. (Not Avoid : 0 – no ; 1 – yes).		0.0794	0.0155	−0.0468	−0.0198	**−0.3232**	**−0.1772**
17. You have a lot of good qualities. (Qualities : 0 – no ; 1 – yes).		**0.3865**	0.0829	**0.3460**	**0.1548**	0.0792	0.0467
18. You have a lot to be proud of. (Proud : 0 – no ; 1 – yes).		**0.4638**	**0.1101**	**0.3552**	**0.1752**	**0.1011**	0.0664
19. You like yourself just the way you are. (Like : 0 – no ; 1 – yes).		**0.4658**	**0.1025**	**0.1586**	0.0716	**0.1938**	**0.1229**
20. You feel like you are doing everything just about right. (Right : 0 – no ; 1 – yes).		**0.4621**	**0.1006**	**0.1290**	0.0580	**0.1968**	**0.1236**
21. When making decisions, you usually go with your ‘gut feeling’ without thinking too much about the consequences of each alternative. (Not Gut Feeling :0–no– yes).		−0.0791	−0.0163	0.0761	0.0291	**0.3367**	**0.1820**
22. You felt lonely. (Not Lonely : 0 – no ; 1 – yes).		**0.4995**	**0.1157**	**−0.3043**	**−0.1454**	0.0022	−0.0001
23. People were unfriendly with you. (Friendly : 0 – no ; 1 – yes).		**0.3744**	0.0783	**−0.2425**	**−0.1053**	−0.0541	−0.0307
24. You feel socially accepted. (Accepted : 0 – no ; 1 – yes).		**0.4297**	0.0892	**0.2242**	0.0984	**0.1172**	0.0708
25. You feel loved and wanted. (Wanted : 0 – no ; 1 –yes).		**0.4701**	**0.1089**	**0.3386**	**0.1635**	0.0693	0.0442
26. You feel adults care about you. (Adults : 0 – no ; 1 –yes).		**0.3798**	0.0857	**0.1259**	0.0574	**−0.1629**	**−0.1049**
27. You feel your teachers care about you. (Teachers : 0 – no ; 1 – yes).		**0.2806**	0.0576	−0.0131	−0.0056	−0.0890	−0.0518
28. You feel your parents care about you. (Parents : 0 – no ; 1 – yes).		**0.3473**	0.0730	**0.1844**	0.0792	**−0.1317**	−0.0805
29. You feel that your friends care about you. (Friends : 0 – no ; 1 –yes).		**0.2730**	0.0532	0.0848	0.0349	**−0.1742**	−0.0966
30. You feel that the people in your family cares about you. (Family : 0 – no ; 1 – yes).		**0.3723**	0.0773	−0.0839	−0.0364	0.0299	0.0171
31. People in your neighborhood look out for each other. (Neighbor : 0 – no ; 1 – yes).		**0.1956**	0.0349	0.0213	0.0087	0.0080	0.0040

**Table 3.** 1994 Resilience's Effect on Smoking, Drinking Alcohol, and Using Illegal Drugs.

**Variables**	Marginal Effect: Smoking								Marginal Effect: Alcohol								Marginal Effect: Illegal Drugs							
	Participation (Probit)				Consumption (OLS)				Participation (Probit)				Consumption (OLS)				Participation (Probit)				Consumption (OLS)			
	Mean	SD	z	p>|z|	Mean	SD	z	p>|z|	Mean	SD	z	p>|z|	Mean	SD	z	p>|z|	Mean	SD	z	p>|z|	Mean	SD	z	p>|z|
**Resiliency Indices:**																								
Overall Index 1	−0.05	0.00	−11.38	***	−0.50	0.06	−7.86	***	−0.06	0.00	−13.46	***	−0.49	0.09	−5.48	***	−0.04	0.00	−15.31	***	−0.18	0.84	−0.22	
Self/Family Index 2	0.07	0.01	14.90	***	−0.25	0.07	−3.49	***	0.06	0.01	13.96	***	−0.05	0.10	−0.45		0.02	0.00	7.11	***	0.10	0.94	0.11	
Self Index 3	−0.01	0.01	−1.38		0.36	0.08	4.44	***	−0.02	0.01	−3.51	***	0.30	0.12	2.58	**	0.01	0.00	2.53	**	0.74	1.11	0.65	
**Policy Measures:**																								
Restrictions	0.15	0.38	0.40		1.13	8.01	0.14		−0.04	0.18	−0.19		−4.80	5.67	−0.85		0.08	0.12	0.59		−37.97	49.39	−0.77	
Program	−0.05	0.04	−1.06		0.91	0.57	1.60		−0.04	0.31	0.14		1.90	7.71	0.25		−0.20	0.12	−1.68		10.51	49.49	0.21	
**Parent Measures:**																								
Parent Smoke/Drink	0.04	0.01	4.05	***	0.19	0.14	1.40		0.00	0.00	8.63	***	0.16	0.07	2.24	*								
Relationship Index	−0.04	0.00	−8.28	***	−0.41	0.07	−6.18	***	−0.03	0.00	−7.22	***	−0.28	0.09	−3.18	***	−0.02	0.00	−8.92	***	−3.33	0.87	−3.83	***
Cig Easy Access	0.10	0.01	11.37	***	1.80	0.12	14.54	***																
Lives w/both parents	−0.06	0.01	−7.96	***	−0.54	0.12	−4.62	***	−0.05	0.01	−6.61	***	−0.31	0.16	−1.93	*	−0.03	0.01	−6.71	***	−2.70	1.65	−1.64	
Mother’s Education	−0.01	0.01	−1.76	*	−0.13	0.10	−1.31		0.01	0.01	2.17	*	−0.26	0.14	−1.88		0.00	0.00	0.10		−0.27	0.20	−1.33	
Father’s Education	−0.02	0.01	−2.40	**	−0.16	0.11	−1.51		−0.02	0.01	−2.45	*	0.11	0.15	0.70		0.00	0.00	0.69		−0.99	1.55	−0.64	
**Peer Measure:**																								
School Peer	−0.05	0.04	−1.06		0.89	0.65	1.36		0.19	0.04	4.57	***	1.16	0.90	1.29		0.06	0.04	1.57		−1.87	12.20	−0.15	
**Demographics:**																								
Age	0.01	0.01	2.02	*	0.58	0.07	8.07	***	0.02	0.01	3.97	***	0.30	0.10	2.96	**	0.01	0.00	3.16	**	−0.66	1.00	−0.66	
Religious	−0.05	0.01	−6.00	***	−1.01	0.12	−8.62	***	−0.06	0.01	−7.59	***	−0.48	0.16	−2.92	**	−0.05	0.01	−9.94	***	−5.49	1.64	−3.35	***
Male	0.02	0.01	2.78	***	0.76	0.11	6.72	***	0.02	0.01	3.14	**	1.41	0.16	8.81	***	0.04	0.01	9.03	***	8.50	1.62	5.25	***
White	0.07	0.01	5.58	***	0.83	0.19	4.31	***	0.11	0.01	8.80	***	0.70	0.27	2.62	**	0.04	0.01	5.91	***	1.00	2.70	0.37	
Black	−0.06	0.01	−4.25	***	−1.68	0.23	−7.34	***	−0.05	0.02	−3.23	***	−0.85	0.33	−2.61	**	0.03	0.01	3.10	**	0.53	3.17	0.17	
Hispanic	0.05	0.01	3.55	***	−0.75	0.19	−3.85	***	0.08	0.01	5.73	***	0.98	0.28	3.55	***	0.04	0.01	4.27	***	−1.31	2.72	−0.48	
Other	0.08	0.03	2.66	**	0.25	0.46	0.55		0.10	0.03	3.20	***	0.98	0.65	1.52		0.06	0.02	2.92	**	6.91	6.19	1.12	
Urban	−0.01	0.01	−0.49		0.31	0.21	1.49		0.00	0.01	−0.09		0.04	0.28	0.14		0.01	0.01	0.56		1.55	2.80	0.55	
Grade	0.02	0.01	3.40	***	−0.18	0.08	−2.25	*	0.04	0.01	6.54	***	−0.14	0.12	−1.09		0.00	0.00	1.11		−0.12	1.07	−0.11	
Fit (OLS regression: adjusted R^2^)					0.1443								0.0550								0.1196			
Fit (probit regression: log likelihood)	−13099								−12520								−7496							
Number	20662				11094				20662				9617				20406				2926			

* p <= 0.05 is statistically significant at 5% level.

** p <= 0.01 is statistically significant at 1% level.

*** p <= 0.001 is statistically significant at 0.1% level.
